# Cocoa Bean Proteins—Characterization, Changes and Modifications due to Ripening and Post-Harvest Processing

**DOI:** 10.3390/nu11020428

**Published:** 2019-02-19

**Authors:** Harshadrai M. Rawel, Gerd Huschek, Sorel Tchewonpi Sagu, Thomas Homann

**Affiliations:** 1 Institute of Nutritional Science, University of Potsdam, Arthur-Scheunert-Allee 114-116, 14558 Nuthetal, Potsdam, Germany; sorelsagu@uni-potsdam.de (S.T.S.); homann@uni-potsdam.de (T.H.); 2 IGV-Institut für Getreideverarbeitung GmbH, Arthur-Scheunert-Allee 40/41, D-14558 Nuthetal OT Bergholz-Rehbrücke, Germany; gerd.huschek@igv-gmbh.de

**Keywords:** cocoa processing, cocoa proteins, classification, extraction and characterization methods, fermentation-related enzymes, bioactive peptides, heath potentials, protein–phenol interactions

## Abstract

The protein fractions of cocoa have been implicated influencing both the bioactive potential and sensory properties of cocoa and cocoa products. The objective of the present review is to show the impact of different stages of cultivation and processing with regard to the changes induced in the protein fractions. Special focus has been laid on the major seed storage proteins throughout the different stages of processing. The study starts with classical introduction of the extraction and the characterization methods used, while addressing classification approaches of cocoa proteins evolved during the timeline. The changes in protein composition during ripening and maturation of cocoa seeds, together with the possible modifications during the post-harvest processing (fermentation, drying, and roasting), have been documented. Finally, the bioactive potential arising directly or indirectly from cocoa proteins has been elucidated. The “state of the art” suggests that exploration of other potentially bioactive components in cocoa needs to be undertaken, while considering the complexity of reaction products occurring during the roasting phase of the post-harvest processing. Finally, the utilization of partially processed cocoa beans (e.g., fermented, conciliatory thermal treatment) can be recommended, providing a large reservoir of bioactive potentials arising from the protein components that could be instrumented in functionalizing foods.

## 1. Introduction

Principal botanical varieties of *Theobroma cacao* L. are Forastero, Criollo and Trinitario. Forastero varieties are regarded as ‘bulk cocoa in trade’ and constitute almost 95% of the cocoa’s total worldwide production [1]. Both the Trinitario and the Criollo varieties produce the ‘fine flavor’ cocoa beans, which account for less than 5% of the total cocoa’s world production [1]. Cocoa protein constitutes 11–13% based on dry weight and may vary depending on geographical origin between 11.8% and 15.7% [2,3]. The average value for the amino acid-based protein content of cocoa bean cotyledons from different varieties was also investigated and lies at approx. 10.4% [4]; for Criollo it lies at 10%, for Trinitario it is between 8.8% and 10.7% and that for Forastero lies at 10.2–11.4% [4]. The value for crude protein (adjusted for alkaloids) is similar to that based on amino protein, although some of the latter values tend to be slightly lower [4]. The average protein content of roasted cotyledons (also termed “nibs”) lies at around 12.5% [1]. Many factors affect not only the quality of proteins such as location (climate, soil, fertilizer, and stress) but also the considered botanical varieties (genomics). In the following, it is initially intended to encompass the extraction, characterization options and classification of cocoa been proteins. In the next step, we address the impact of different stages of cultivation and processing with regard to the induced changes in the protein fractions. Special focus is laid on the major seed storage proteins (vicilin and albumins) throughout the different stages of processing.

## 2. Extraction and Classification of Cocoa Proteins

Some of the early attempts to extract proteins from cocoa beans were conducted after the removal of lipids (soxhlet extraction with ethyl ether) and of phenolic compounds with methanol followed by extraction with buffering solutions containing different additives (acidic pH conditions using acetic acid, urea, hexadecyltrimethylammonium bromide, ascorbic acid, and sodium ethylenediaminetetraacetate (EDTA)), resulting in a maximum recovery of 25% of the protein nitrogen [5,6]. The extracted proteins are thereafter classified according to their solubility characteristics originating from the concept of T. B. Osborne (1859–1929) in the following manner: distilled water delivers the albumin or water-soluble proteins, a diluted salt solution to obtain a globulin fraction, extracted with 70% aqueous ethanol followed by 0.2% NaOH, yielding prolamine and glutelin fractions. Accordingly, 32–37% albumins, 19–25% globulins, 11–13% prolamines and 30–37% glutelins are allocated to non-pigmented cocoa bean varieties. Similarly, 51–71% albumins, 1–25% globulins, 12–20% prolamines and 8–12% glutelins are allocated to pigmented cocoa bean varieties, bearing in mind that only a partial protein recovery is determined [6]. The problems associated with discoloration and protein insolubility resulting in poor recovery are believed to be caused primarily by residual polyphenolic materials not removed by the preceding methanol extraction [6]. These protein–phenol interactions can be classified into two subgroups: non-covalent and covalent interactions [7]. Principally, three potential types of non-covalent interactions of phenolic compounds and proteins have been suggested: hydrogen, hydrophobic, and ionic bonding [7]. The phenolic compounds are also susceptible to both enzymatic and non-enzymatic oxidation in the presence of oxygen, leading to reactive and redox active *o*-quinones, an electrophilic species, capable of undergoing a nucleophilic addition to proteins [7]. This results in the derivatization of protein-bound amino acids invoking consequently also cross-linking reactions. Both of these two types of complexing interactions results in an increase in protein aggregation, insolubility and discoloration [7].

In the following years, the studies of Voigt et al. (1991–1997) were decisive in improving the protein extraction and characterization especially in the context of their role while analyzing the biochemical aspects of cocoa bean fermentation [8,9,10,11,12,13,14,15,16,17]. In the same decade, cDNAs encoding of the major albumin and globulin of cocoa seeds was achieved and the proteins were consequently cloned and sequenced [18,19,20,21]. The reported amino acid sequence of the albumin was homologous with the Kunitz protease and α-amylase inhibitor family [20,21]. Based on amino acid sequences, subunit compositions, and the processing of the corresponding polypeptide precursors, the globulins can be assigned to the vicilin-like globulins of storage proteins, previously found only in legumes and cotton [18,19]. In the cocoa beans, vicilin is synthesized (partly similar to that in cottonseed) as an approx. 70 kDa molecular weight precursor protein and then processed to 47 kDa and 31 kDa mature proteins [18]. A latter study documents that the entire cocoa vicilin is encoded by a single gene and that heterogeneity of the vicilin subunits may be attributed to statistical post-translational modifications [22]. In contrast to the major albumin and the vicilin class globulin, there are, however, at this stage no data available concerning prolamin and the glutelin which were also found in the seeds of *Theobroma cacao* [9].

**Figure 1:**
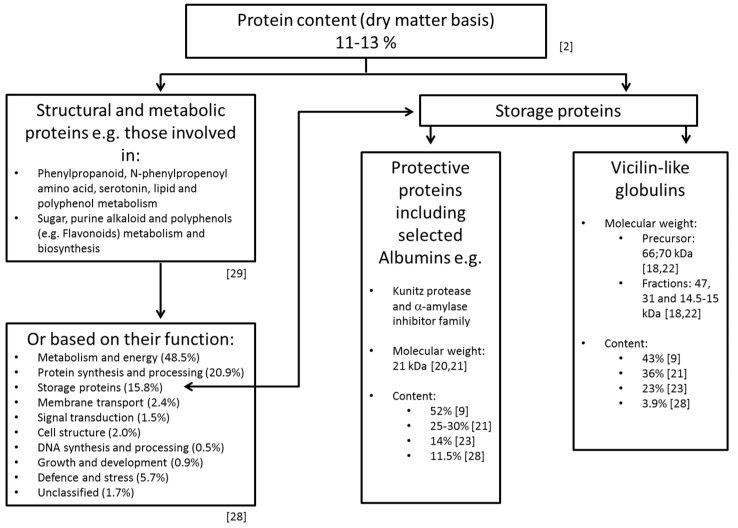
Contemporary classification of cocoa seed proteins [2,9,18,20,21,22,23,28,29].

Most of the cocoa seed proteins are solubilized thereafter while working with a dry polyphenol-free acetone powder and high-salt buffer systems. The separation of albumins is, for example, also achieved by the following desalting process while applying dialysis against a salt-free buffering solvent [22]. The major proteins of cocoa beans allocated to vicilin and albumin classes thereafter represent about 43% and 52% of the total cocoa seed proteins, respectively [9]. Other studies report that vicilin constitutes ca. 23% and the albumins constitute around 14% of the total soluble seed proteins [23]. The observed discrepancies in the values are most likely dependent on the extraction procedure and allocation method used. Both studies applied previously treated material with ice cold acetone to remove the interfering polyphenols. In the first study, the results are based on proteins which were extracted and fractionated into various solubility classes by using different buffering systems [9]. The protocol applies successive extractions with 10 mM Tris-HC1 (pH 7.5, containing 2 mM EDTA), 0.5 M NaCl (containing 2 mM EDTA and 10 mM Tris-HCl, pH 7.5), 70% (*v*/*v*) ethanol and 0.1 M NaOH, to obtain the albumin, globulin, prolamin and glutelin fractions, respectively [9]. The protein contents of these extracts deliver corresponding data reported. In the latter study [23], the proteins are extracted to be compatible with the high-resolution technique of immobilized pH gradient of the two-dimensional electrophoresis (2-DE). For this purpose, a solubilization solution containing different additives (3% (*w*/*v*) CHAPS (3-((3-cholamidopropyl) dimethylammonio)-1-propanesulfonate), 8.5 M urea, 0.15% (*w*/*v*) DTT and 3% (*v*/*v*) carrier ampholytes in the pH range of 3–10) can be recommended. The evaluation of 2-DE data deliver the 47, 31 and 15 kDa vicilin-type storage protein components [18,22] representing 23.1% of the soluble seed proteins for globulins, and 14.1% of the 21 kDa trypsin inhibitor for albumins, respectively [23]. These values are also lower than those reported (36% for globulin storage protein [24] and between 25% and 30% for the trypsin inhibitor [21]). The discrepancy was assumed by the authors [23] to be based on the electrophoretic methods used (estimation by SDS-PAGE being higher and since the polypeptide bands evaluated may contain different individual species). The most common gel-based technique used in a proteomic laboratory is 2-DE [25]. Protein quantities, as well as their profiles derived from two-dimensional gel electrophoresis, show striking differences for non-fermented cocoa beans, depending on their geographical origin [26]. Although ‘Osborne fractionation’ is still widely used, it is more usual today to classify seed proteins into three groups: storage proteins, structural and metabolic proteins, and protective proteins [27]. Figure 1 documents a tentative classification of the proteins based on different studies. Additional proteins in the cocoa powder after buffer extractions can be extracted with a solution containing chaotropic agents [28].

**Figure 2:**
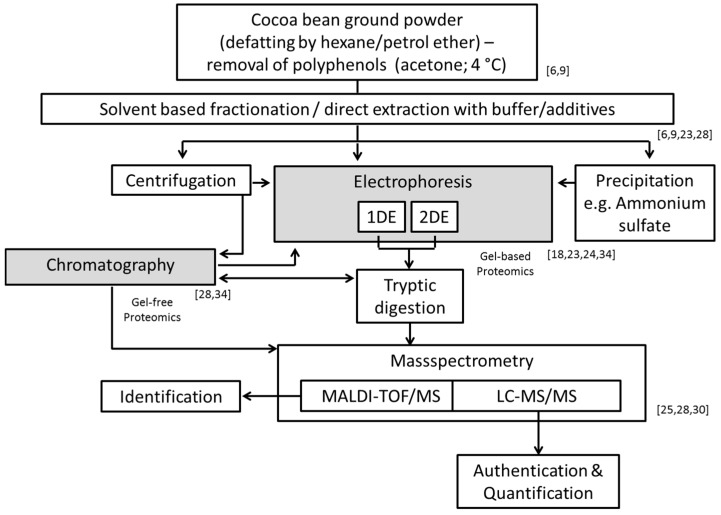
Compilation of the methods and characterization options applied for cocoa bean proteins denoted with corresponding relevant studies [6,9,18,23,24,25,28,30,34].

During recent years, the area of proteomics has undergone rapid developments integrating high-resolution, fast, stable and accurate mass detection. Proteomic technologies have made an extensive development with the discovery of different protein ionization methods, notably the electrospray ionization (ESI) and matrix-assisted laser desorption/ionization (MALDI) techniques in mass spectrometry (MS), which enable proteins to be identified [25]. MALDI-time of flight/MS (MALDI-TOF/MS) is also employed to characterize the water-soluble portion of the proteomic seed extracts from different varieties (Forastero, Criollo, and Trinitario) of *Theobroma cacao*. Most of the proteins detected with this approach show molecular weights between 8 and 13 kDa, while a cluster at 21 kDa is attributed to albumin [30]. The development of gel-free proteomics (while using liquid chromatography (LC), and capillary electrophoresis) provides here an excellent alternative to more accurately quantitating protein and enabling deeper explorations of complex proteins [25,31]. Both the gel-based and gel-free methods integrate MS for protein profiling, protein identification (with prior tryptic digestion, and analysis of the digestion products), and quantification, as well as analysis of protein modifications and interactions [25]. Both gel-based and gel-free approaches have also been developed and utilized in a variety of combinations to separate proteins from tissue culture of cocoa beans prior to mass spectrometric analysis [32,33]. In this context, one of the first attempts to characterize the whole cocoa bean proteome by nano-LC-ESI MS/MS analysis using tryptic digests of cocoa bean protein extracts has recently been made indicating that more than 1000 proteins can be identified while applying a species-specific *Theobroma cacao* database [28]. Most of these are related to metabolism and energy household, protein synthesis and processing and response to different stress stimuli or connected with defense scenarios [28]. Vicilin and albumin are classified as storage proteins and show again the highest abundance among all detected proteins, although compared to the total protein amount their relative amounts are only 3.9% and 11.5%, respectively [28]. These levels are lower than the values discussed above of between 43% and 23% for vicilin, between 52% and 14% for albumin [9,23]. It is likely that again the discrepancies could arise from inefficiency of selective solubilization of proteins [9] and overestimation, due to detection insufficiency based on electrophoretic methods and eventually also due to incomplete tryptic digestion preceding the nano-LC-ESI MS/MS analysis. Figure 2 gives a summary of the currently applied methods for extraction, separation, protein allocation and identification.

For the purpose of cocoa species verification, the extracted proteins and the individual peptides resulting from their tryptic digestion are initially controlled in silico (e.g., using software tools such as Skyline software [35]) with the BLAST algorithm (Basic Local Alignment Search Tool from the database; UniProt—the Universal Protein Resource: http://www.uniprot.org/) as described in [36,37]. The identification then proceeds with help of mass-spectra algorithms and search engines (e.g., Mascot, Matrix Science, London, UK; www.matrixscience.com) and available databases [28]. In order to assess whether searching different databases would yield a higher number of cocoa-specific protein hits, searches using different databases with taxonomy *Viridiplantae*, and custom databases containing only *Theobroma cacao* entries have been recommended [28]. The recently published cocoa genome sequences can also be useful to create a predicted proteolytic fragment database [32,38,39,40]. In this context, the Universal Protein Resource (UniProt) is the most comprehensive database/resource for specific protein sequences and annotation data (http://www.uniprot.org/). A recent search in this database confirmed at least 40,964 (compared to 40,941 according to [28]) *Theobroma cacao* protein entries (visited on 4th January, 2019), of which only six were reviewed representing records with information extracted from literature and curator-evaluated computational analysis. In comparison, based on this background and utilization of the available sophisticated MS tools, both gel-based and gel-free approaches have been more frequently applied for proteome analysis during zygotic and somatic embryo maturation, in order to identify alterations in protein abundance that correlate with maturation of cocoa embryos or with the intention to accelerate breeding programs and plant development [25,32,33,41,42,43,44,45,46].

In summary, it appears from the state of art that cocoa proteins need to be better accessed, especially while comparing the contents of different varieties of *Theobroma cacao.* The discrepancies in protein content observed in the reviewed literature need to be eliminated to obtain more consistent data. The methodical approach using bioinformatics algorithms, targeted peptide biomarkers and high-resolution MS can be recommended here for authentication of the analyzed proteins [36,37,47], especially while considering the different aspects of post-harvest processing.

## 3. Changes in Protein Composition during Ripening and Maturation of Cocoa Seeds

**Figure 3:**
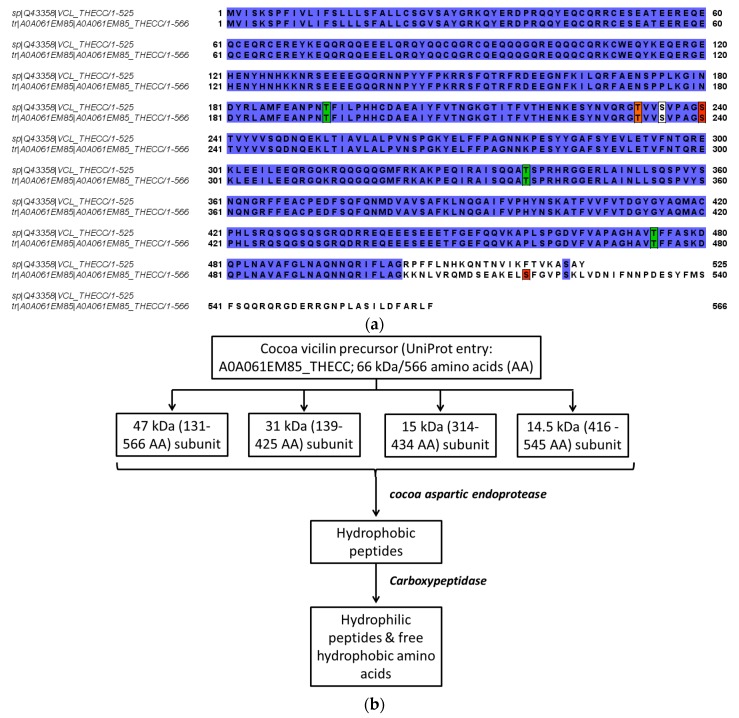
(**a**) Comparison of two entries of the storage protein vicilin from *Theobroma cacao* (a reviewed and a non-reviewed versions) from the database UniProt (http://www.uniprot.org/; 28th January, 2019) showing the similarities (blue) in the amino acid sequence. Positions for predicted post-translational modifications [50] are marked: phosphorylation (red; at positions 232 (Thr), 235 (Ser), 240 (Ser), and 518 (Ser)), or glycosylation (green; O-GLcNAc modifications at positions 193 (Thr), 235 (Ser), 338 (Thr) and 474 (Thr)). The common residue (white, 235 (Ser)) serves as a possible site for both modifications. (**b**) Postulated degradation of cocoa vicilin precursor during maturation by endogenous enzymes and fermentation adapted from [10,11,12,22,57]; the 47 kDa subunit may only encompass the sequence 131–545 amino acids [50]. Specific sites for phosphorylation or glycosylation of the subunits are given in Figure 3a [50]. AA represents the amino acid sequence of the subunits.

**Figure 4:**
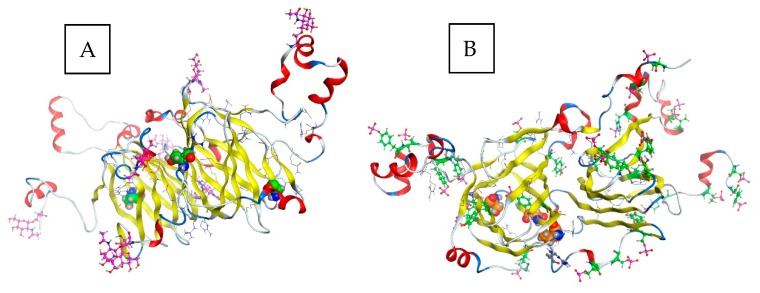
Homology modeling of the vicilin storage protein. (**A**) Postulated glycosylation (O-GLcNAc) and (**B**) phosphorylation sites (both in pink) in the storage protein vicilin from *Theobroma cacao* (entry: Q43358; UniProt: http://www.uniprot.org/; 28th January, 2019). The data indicate that the modifications (illustrated in green/orange spacefilling model) as reported in [50] are most probably occurring after the proteolytic processing of the 66 kDa precursor [9,22]. Please see supplementary information provided.

Cocoa pods need 4–5 months to grow to full size following pollination and the beans contained therein have reached the maximum development, thereafter they are allowed to ripen for approximately 4 weeks [5]. The major textural changes that cause softening of fruit during the ripening (and proceeding in the fermentation process) result from enzyme-mediated alterations in the structure and composition of the cell wall and partial or complete solubilization of cell wall polysaccharides, such as pectins [48]. During ripening, the mucilaginous pulp surrounding the beans undergoes changes critical to a successful fermentation, primarily resulting in an increase in fermentable carbohydrate components [5,49]. Data on changes in the amount of seed storage proteins during ripening and maturation of cocoa beans are sparse. During the ripening of cocoa fruit, both the amounts of the total and extractable protein content of seeds decreased by 25% or 19% respectively, but no consistent qualitative trends were not apparent [5]. Some differences in solubility trends between the investigated different ripening stages (135–160 days postpollination) afterwards were noted, although the amino acid analysis did not reveal any specific related differences [5]. One of the earliest studies to report on the accumulated globular storage protein during seed development/ripening was achieved while using antibody against a large subunit of the vicilin (7S) globulin, also capable of cross-reacting with all of the 7S globulin subunits [16]. The vicilin-like globulin of the cocoa seeds contains two prominent subunits with apparent molecular masses of 47 kDa and 31 kDa and three smaller polypeptides with apparent molecular masses of 15.5 kDa, 15.0 kDa, and 14.5 kDa arising from the proteolytic processing of a 66 kDa precursor [9,22]. Further data indicate that two 28 kDa and 16 kDa components also observed during characterization of vicilin are not intrinsic subunits of the cocoa vicilin but are generated by partial proteolysis during preparation of this particular globular storage protein, their content being strongly diminished while working with a sufficiently high concentration of pepstatin A, an inhibitor of aspartic endoproteinases [9,22]. These findings are derived from the combined results of the epitope mapping of the corresponding polyclonal antibody, sequence coverage as documented by the MALDI-TOF/MS mapping of the tryptic hydrolysates of the purified fractions and the amino acid profiling/kinetics of the release of amino acids by carboxypeptidase Y [22]. A comparison of two entries (60.8 kDa/525 amino acids reviewed and non-reviewed 65.5 kDa/566 amino acids version) from the database UniProt for cocoa vicilin showing similarities in the amino acid sequence are depicted in Figure 3a. In the same context, the results reported in [22] are related to the non-reviewed entry (A0A061EM85_THECC; 65.5 kDa/566 amino acids). Correspondingly, Figure 3b shows the predicted proteolytic processing of the vicilin precursor based on this particular sequence [22]. The localization of the 47 kDa vicilin based on the peptide fragments may differ as reported in the context of further studies [50]. Amino acid position 545 represents the C-terminal end-point of the 47 kDa subunit, as no peptide was obtained derived from the amino acid sequence beyond position 545 (Figure 3a,b). This result suggests that the absolute C-terminus of the 566-amino-acid-long vicilin precursor sequence undergoes a swift carboxypeptidase attack leaving no peptide(s) but just free amino acids or di- and tri-peptides behind [50]. Finally, the two-dimensional gel electrophoresis of these mature subunits of cocoa vicilin also revealed their heterogeneity, postulated to result from post-translational modifications of various amino acid side chains, e.g., due to the action of a protein deaminase during maturation [22]. A more recent study complements these previous findings and documents that the vicilin storage protein subunits may undergo phosphorylation (modification at positions 232 (Thr), 235 (Ser), 240 (Ser), and 518 (Ser)), or glycosylation (O-GLcNAc modifications at positions 193 (Thr), 235 (Ser), 338 (Thr) and 474 (Thr)); please also refer to Figure 3a [50]. Figure 4 shows, for the first time, the tentative modelling of the storage protein vicilin from *Theobroma cacao* (entry: Q43358; reviewed version from the database UniProt: http://www.uniprot.org/; 28th January, 2019), visualizing the accessibility of the amino acid residues for the post-translational modifications. The molecular modeling is based on the methods described in [51] (please also refer to Supplementary Figures S1–S3 and Table S1 provided). The model also shows the different theoretical sites for glycosylation (Figure 4a) or phosphorylation (Figure 4b). The homology modeling data further indicate that the phosphorylation and glycosylation sites as reported in [50] are buried within the molecule and are most probably accessible after the initial proteolytic processing of the 66 kDa precursor [9,22] as discussed above (please refer to supplementary information provided).

Finally, a major 43 kDa protein also present in the non-fermented bean sample could be identified belonging to protein kinase superfamily [50]. The most prominent protein with a molecular weight of 21 kDa is again allocated to albumin by tryptic digestion and MALDI-TOF/MS analysis [50].

Further studies address specific enzymes [52,53,54,55,56]. Acyl-thioesterase activity has been examined at two developmental stages (105/130 days postanthesis) in cocoa documenting low and high stearate productions [54]. A further study addresses a cysteine protease expressed during the process of the maturation of the cocoa seed [52]. This enzyme is part of the defense mechanism induced in response of the action of the parasite *Moniliophthora perniciosa*, a fungus that causes witches’ broom, which is one of the diseases that most affects the production of cocoa, dramatically reducing crop yields.

Cocoa beans also contain peroxidase, a heme-containing oxidoreductase, which efficiently oxidizes the phenolic molecules using H_2_O_2_ as a co-substrate. An increase of peroxidase activity in the seeds of cocoa during their ripening has been documented. The major cocoa isoperoxidase is an acidic enzyme with the isoelectric point (pI) of 4.7, together with two basic isoenzymes of the peroxidase with pIs of 8.6 and 9.0 detected, respectively, during the process of the fermentation [55]. The activity proceeds to increases further (about 10 times) during the fermentation and drying of the beans, again contributing to sensory perception of cocoa [55]. The role of these enzymes is important, since the oxidation products of phenolic compounds may not only modify amino acids, peptides and proteins [7], but also is determining in reducing the astringent and bitter taste, thus also contributing to flavor of cocoa [55]. In a similar context, a series of N-phenylpropenoyl amino acids categorized as multifunctional polyphenol derivatives (with aspartic acid amide of caffeic acid-(30,40-dihydroxy-(E)-cinnamoyl)-L-aspartic acid as the most abundant constituent) are identified as key contributors to the astringent taste of non-fermented cocoa beans [58,59] with their content decreasing during the cocoa seed development [29]. The results contradict with those reporting an increased accumulation in the advanced stage of seed development [60].

In *Theobroma cacao* seeds, an aspartic proteinase has been proposed to be a key enzyme involved in the formation of one group of cocoa aroma precursors [53]. At least two distinct aspartic proteinase genes (TcAP1 and TcAP2) are expressed during early cocoa seed development. Of these, the corresponding TcAP2 protein has been proposed to be primarily responsible for the majority of the industrially important protein hydrolysis that occurs during cocoa bean fermentation [53]. Aspartic endoproteinase activity increases rapidly during embryo expansion, reaching a maximal activity before final maturity, a prerequisite for the degradation of seed storage proteins during the following post-harvest processing [61]. Voigt et al., in 1995, showed that this enzyme accumulates with the vicilin-class globulin during bean ripening [16]. The enzyme consists of two peptides (29 and 13 kDa) that have been proposed to be derived from a single 42 kDa precursor zymogen, possibly by self-digestion [9,53]. The mature cocoa seed aspartic proteinase has been proposed to cleave protein substrates between hydrophobic amino acid residues producing oligopeptides with hydrophobic amino acid residues at their carboxyterminal ends [9,53]. The enzyme activity is shown to be optimal at pH 3.5 and is inhibited by pepstatin A. A recent check-up for aspartic proteinase in the protein database UniProt (http://www.uniprot.org/; 17.01.2019) delivers up to 107 *Theobroma cacao* entries, largely based on differing gene ontology (including the genes *AP1* and *AP2*) and representing different isoforms.

Finally, many reports encompass changes in protein expression during zygotic and somatic embryo maturation [32,43,44], where the seed storage protein is more strongly accumulated in cocoa zygotic embryos compared to that in their somatic counterpart [32]. The identified proteins represent an array of functional categories, including seed storage, stress response, photosynthesis and translation factors [32]. A system level analysis of cocoa seed ripening further revealed the accumulation of proteins and metabolites involved in biotic and abiotic stress resistance, leading to e.g., polyphenol accumulation [62], but also covered the interplay of different specific primary and secondary metabolism pathways important for the major compound classes (e.g., lipid and sugar metabolism as well as those of selected secondary bioactive plant metabolites such as alkaloids and polyphenolic compounds) involved in cocoa aroma and health benefits [29,62,63]. Accordingly, at the stage of reserve accumulation phase of cocoa seeds [29], most of the amino acids reach their lowest level similar to the trend observed for the reserve accumulation period of *Arabidopsis thaliana* seeds [64].

## 4. Changes in Proteins during Post-Harvest Processing

The complex composition of cocoa bean flavor has been discussed depending on bean genotype, post-harvest treatments such as pulp pre-conditioning, fermentation and drying, and industrial processes such as roasting as well as the type of soil and age of cocoa tree [65]. However, how the age of the cocoa tree and soil chemical compositions influence the formation of flavor precursors still remains unclear [65]. In this context, the impact of the farming system, the ripeness state of the pods, and the role of microbial interactions on the fermentation has also been evaluated [1]. Post-harvest processing on farms and plantations involves the following four main steps of pod opening and beans removal from the pod, beans fermentation, and drying. These steps are most likely also responsible for many post-transitional/post-processing modifications occurring to different proteins [1]. Consequently, the most effective and essentially critical step-hear appears to be that of fermentation which determines the development of flavor quality attributes of the commercial cocoa beans [1]. Most of the recent studies have been directed to enlighten the role of fermentation as a quality determining steps for the following roasting process [26,50,57,65,66,67,68,69,70]. Early studies of Voigt et al. in the 1990s confirm that cocoa seed storage proteins play an important role in flavor development since essential precursors of the cocoa-specific aroma components are formed from their degradation during the fermentation process [9,10,11,12,13,15]. Their experimental approach showed that these aroma precursors are released from seed proteins by the dual activity of the already mentioned aspartic endoprotease and a carboxypeptidase (Figure 3b) [10]. Their studies also document the proteolysis products obtained when these proteins are subjected to autolysis at pH 5.2, under conditions of optimal fermentation consisting of both hydrophilic peptides and hydrophobic free amino acids (Figure 3b), as also observed by other studies [53]. This specific mixture of hydrophilic peptides and hydrophobic free amino acids is capable of producing the typical cocoa flavor when roasted in the presence of reducing sugars and deodorized cocoa butter [10]. On the basis of sensory evaluation of the resulting aromas by sniffing analysis, it was further demonstrated that the fraction of hydrophilic peptides generated in vitro contains the essential cocoa-specific aroma precursors necessary for the following complex Maillard reaction under these conditions. In comparison, the patterns of free amino acids alone, specifically leucine, alanine, phenylalanine, and tyrosine found in fermented cocoa seeds, do not contribute towards the formation of this typical cocoa aroma under roasting conditions in vitro [10]. However, they are still likely to react with the reducing sugars fructose and glucose during the Maillard reaction as implicated in the following works [71].

To our present knowledge, the fermentation, which is essentially controlled by proteolytic activity within the cocoa bean, is also driven by changes in the presence of fermentation by-products as a result of microbial activity outside the bean [67]. This is so-called “post-mortem proteolysis” and therefore depends on the variable/desired pH value (4.0–5.5) in the seed, e.g., established by acetic acid absorbed from the fermenting pulp [72]. An aseptic artificial fermentation system, free from microbial activity, has also been reported, capable of simulating the proteolytic degradation of cocoa proteins as observed during commercial fermentation, where again acidification was the most crucial parameter for the protein degradation [67]. The changes occurring to vicilin-like storage protein at different stages of fermentation (from the non-fermented stage up to the dried cocoa beans) have been addressed in detail [50]. Analysis of vicilin breakdown peptide pool during fermentation by the UHPLC-ESI-MS/MS revealed an initial increase and subsequent decrease in the diversity of peptides with an increasing degree of fermentation [50]. The results also indicated that the sequences of free peptides are localized to distinct zones spread throughout the entire C-terminal vicilin sequence, except the N-terminal where no peptide hits could be found for the amino acid sequence 1–131 [50]. There are no detectable peptides found in the fully fermented and dried bean samples. This observation in turn shows the contribution to the pool of free amino acids or di- and tri-peptides as potential conjugative moieties for aroma compounds, such as Amadori or Strecker reaction products during the following roasting process [50,73]. The most abundant albumin storage protein is steadily and homogenously degraded without forming breakdown intermediates with a molecular weight larger than approximately 10 kDa [50].

The variability in the peptide pattern was observed among cocoa samples of different geographical origins, suggesting diversified proteolytic activities could be a relevant feature in this context [69]. The applied combination of proteomic and peptidomic fingerprinting enables a more comprehensive analysis of the attributes that characterize storage protein degradation in cocoa during microbial fermentation [26]. This study also confirms that the major differences in protein content of non-fermented cocoa beans are predominantly attributed to the geographic origin in terms of continental regions. The authors also attest that the formerly detected diversity of peptides could not be correlated to the geographical origin but rather to the degree of fermentation, depending on the fermentation method applied in the country of origin underlining again the role of diversified proteolytic activities [26]. In this context, more than 800 unique oligopeptides, excluding di- and tri-peptides, documenting the largest collection of cocoa oligopeptides, have been identified and relatively quantified by utilizing UHPLC-ESI-quadrupole-quadrupole-time-of-flight (Q-q-TOF) mass spectrometric analysis. In the same context, more than 800 fermentation peptides could recently also be unambiguously identified, providing unprecedented mechanistic details of cocoa fermentation [74]. The cocoa-specific aroma precursor fractions have also been characterized by MALDI-TOF and their amino acid sequences determined by ESI-MS/MS, allowing for a partial purification and a consequent detailed characterization of these peptides responsible for the generation of the cocoa-specific aroma components [66].

Polyphenols are also oxidized by polyphenol oxidase during fermentation and drying which reduce the astringency and bitterness of the beans, thus enhancing the flavor of cocoa beans [65]. The total amount of polyphenols in dried fresh cocoa beans may vary between 12% and 20% (*w*/*w*) and these are responsible for its high astringency, contributing to their bitterness as well [1]. Three main groups of polyphenols are present: anthocyanins, flavan-3-ol (catechins), and proanthocyanidins, corresponding to approximately 4%, 37%, and 58%, respectively [60,75,76,77,78,79]. An increase of peroxidase activity in the seeds of cocoa during their ripening and stronger during fermentation has been observed, implicating a possible oxidation of these compounds [55,65]. In a similar context, the polyphenols (depending on their structure) are also readily oxidized by polyphenol oxidase [7]. The combined effect of the interaction of the oxidized phenolic compounds with the degraded protein products (amino acids, and peptides; Figure 3b) during post-harvest processing may result in a further modification of the protein-based aroma precursors, although detailed studies in this respect, e.g., those similar to modification of coffee bean proteins [7,80,81], have not been addressed.

The vicilin-class globulins are quantitatively degraded during fermentation (88–90% of the initial content), providing the cocoa-specific aroma precursor fractions for the following Maillard reactions during drying and roasting [9,65,68,71,73,82,83]. The compounds produced in the complex interactions during the thermal mediated Maillard reaction comprise nitrogen and oxygen heterocyclic compounds, aldehydes and ketones, esters, alcohols, hydrocarbons, nitriles and sulphides, pyrazines, ethers, furans, thiazoles, pyrones, acids, phenols, imines, amines, oxazoles, and pyrroles [65]. Approximately 600 flavor compounds have been identified from cocoa beans and cocoa products [65,84]. The complex formation of cocoa and chocolate flavor is discussed in detail in [65,71,85]. It is influenced by different factors starting with the composition of the beans, post-harvest treatment (e.g., fermentation, and drying), processing (e.g., roasting encompassing the Maillard reactions and alkalization to change color) and eventually further fine-tuning during chocolate manufacture (conching). Depending on whether the proteolytic activity has been more or less intense during the fermentation, the result in the color development during the drying step will also be different. Similarly, depending on whether the fermentation will be more or less intense (more or less consumption of reducing sugars), the proportion of residual reducing sugar will also influence the development of the color from the resulting Maillard reaction products during drying and roasting. The relevant processes inducing the changes in the proteins fractions during post-harvest treatments are summarized in Table 1.

## 5. Bioactive Potential Arising from Cocoa Proteins

The health benefits of cocoa and cocoa-based products have been reviewed, and its potential for prevention/treatment of allergies, cancers, oxidative injuries, inflammatory conditions, anxiety, hyperglycemia, and insulin resistance has been discussed in detail [89]. Most of these positive evaluated effects have been correlated with the high content of the different secondary plant metabolites present especially encompassing the group of polyphenolic compounds, where several mechanisms have been proposed that might confer cocoa’s possible health benefit [90,91,92,93]. Exemplarily, being a rich source of flavonoids, cocoa represents a group of potent antioxidant and anti-inflammatory agents, documenting benefits for cardiovascular health [91,94,95,96]. Cocoa flavanols are also reported to have neuromodulatory, neuroprotective and antidiabetic actions in humans [45,97]. In this context, much recent work has been directed to elucidate the effect of processing on antioxidant properties of cocoa and cocoa products [98,99,100,101].

The bioactivity derived from intact cocoa proteins, or their degradation products and/or their derivatives, e.g., resulting from post-harvest processing, has been only sparsely addressed. A good example illustrating the biological activity of the intact proteins is that on cysteine protease of *Theobroma cacao* against witches’ broom as already mentioned in the preceding section [52]. The utilization of such activities can be used for developing new products of commercial interest [52]. The formation of small peptides in fermented cocoa beans offers a large reservoir for other interesting aspects, since small peptides have been discussed as compounds imparting health benefits [26]. Food-derived bioactive peptides represent one such source of health-enhancing components, which can be released during gastrointestinal digestion or food processing. They can be physiologically active, either in the native protein state or as products of hydrolysis in vivo or prior to consumption [102]. Bioactive peptides usually contain 3–10 amino acid residues; their activity is based on their amino acid composition and sequence [103] which include several regulatory mechanisms related to nutrient uptake, immune defense, antioxidant, neuroprotective and antihypertensive properties [104,105,106,107]. Peptides which are not degraded in proteolysis can theoretically be absorbed intactly. It has been suggested that dipeptides and tripeptides are absorbed in the intestine [108,109]. Furthermore, it has also been reported that tripeptides containing a C-terminal proline–proline bond are usually resistant to human proteolytic enzymes [110,111].

In this context, a recent study reported the presence of a bioactive peptide (DNYDNSAGKWWVT) from a hydrolyzed cocoa by-product that was found to have antioxidant property, which could be used therapeutically for the prevention of age-related diseases [112]. The study documents that the peptide protects *Caenorhabditis elegans* from oxidative stress and is responsible for the modulation of synaptic and proteosomal functions. The peptide originates from the storage albumin fraction (21 kDa seed protein), a trypsin inhibitor from *Theobroma cacao* as confirmed by blast analysis in the protein database (https://www.uniprot.org; 28th January, 2019; entry: P32765). Similarly, antitumor activity was also observed in the albumin fraction, which inhibits the growth of cells in murine lymphoma, documenting one of the earliest reports on biological activity of semifermented-dry cocoa protein fractions [113]. The activity could be attributed to its hydrophobic and sulfur amino acids profile that confers antitumor and antioxidant potential. Free radical-scavenging capacity was also observed mainly in the albumin and glutelin fractions from cocoa [113,114]. Finally, the albumin fraction also shows antitumor activity in a mouse murine model of lymphoma L5178Y, indicating that it could be considered as a source of potential antitumor peptides [107]. Antioxidant and angiotensin-converting enzyme (ACE) inhibitory (anti-hypertensive) activities of cocoa autolysates after removing the partly interfering fat, alkaloids and polyphenols have also been elucidated, conferring again another attribute contributing to its health-promoting properties [115]. A recent review discusses on a possible antimicrobial potential of cocoa bean shell related to a diverse pool of bioactive compounds with antimicrobial properties including, beside other compounds, phenolics and bioactive peptides [116]. A further study suggests that cocoa products originating from different post-harvest processing steps may possess mild dipeptidyl peptidase-IV inhibitory activity, and that processing steps such as fermentation may actually enhance inhibition activity [117]. While considering the anti-obesity and anti-hyperglycemic effects, one potential mechanism relates to the inhibition of dipeptidyl peptidase-IV. Glucagon-like peptide-1 is a hormone, which is rapidly degraded by dipeptidyl peptidase-IV. The hormone also stimulates insulin release in response to glucose ingestion, increases satiety, and slows gastric emptying [117]. The compounds responsible for dipeptidyl peptidase-IV inhibition, according to the authors, may represent a previously uncharacterized pool of dietary bioactives beyond the flavanols and flavanol products and still remain to be elucidated [117]. While reducing native polyphenols, fermentation simultaneously produces complex polyphenol oxidation products, which may interact with proteins, peptides and amino acid components [7]. These non-native products may retain some polyphenol structure and activity and/or introduce potentially new activities [117].

Cocoa beans also contain a further interesting group of compounds arising from the enzymatic and/or (chemical) decarboxylation of amino acids representing bioactive amines, where mainly 2-phenylethylamine, tyramine, tryptamine, serotonin and dopamine are found [118,119,120]. Cocoa can also be a source of polyamines (spermidine and spermine), which may also contribute to cocoa’s antioxidant activity [118]. The biogenic amines play relevant roles in plant development and human health and can be formed during fermentation [118]. At low levels, bioactive amines are positively correlated to human health; however, some amines, at high levels, may cause adverse effects to human health [118]. The changes in bioactive amines may be partly attributed through amino acid decarboxylation by microbial enzymes during the fermentation process of cocoa beans and their fate has been discussed in [118]. The roasting process also modifies significantly the profile and levels of biogenic amines [120].

Cocoa products undergo several steps of thermal treatment (drying/roasting) during processing where Maillard reaction products originating from the interactions of proteins, peptides and amino acids with reducing sugars, classified as early-stage “Amadori products” or advanced brown pigments termed “melanoidins”, are formed. Melanoidins are brown, high-molecular-weight products of Maillard reaction [121] and may also contribute to the radical-scavenging potential [122]. Further, it has been suggested that complex polyphenol oxidation and condensation products may also be integrated in melanoidins [117]. Recent studies also discuss the loss of the naturally occurring antioxidants (flavonoids), while others, such as Maillard reaction products, are formed while considering the different stages of cocoa processing [99,123]. While comparing raw, pre-roasted and roasted cocoa samples, increased radical-scavenging activity and reduced growth of pathogenic bacteria in different molecular weight fractions (>30, 30–10, 10–5 and <5 kDa) of roasted cocoa were determined [122]. However, the study also documents that also beneficial bacteria are suppressed in their growth activity [122]. The structure and biological activities of melanoidins (antioxidant, antimicrobial, anticancer, antihypertensive, cytotoxic, genotoxic, and detoxifying activities) have recently been comprehensively reviewed, giving some implications for human health [121,124]. These compounds are only partially characterized and their activities are poorly understood, but provide a potential reservoir for novel and potent bioactivities, underlining the need for further research in this area [117,121,122,124]. Some very recent works not only underline the complexity of this compound class, but also reveal that the high-molecular-weight melanoidin fractions formed may integrate phenolic compounds during roasting, contributing correspondingly to their antioxidant activity [86,87,88]. Finally, the protein crosslinking via the Maillard reaction has been shown to alter the functional properties of several food proteins, but the potential to use this chemistry to alter the functional performance of cocoa proteins has yet to be fully explored [125]. In summary, Table 2 documents the biological activity of the most relevant protein and their modified fractions.

## 6. Conclusions

In conclusion, the present study reviews the literature on the impact of different stages of cultivation and processing with focus on the changes induced in the protein fractions. It surely does not handle all the publications available in the field, but should prove helpful for these researchers needing a quick start in this particular field of research. Some of relevant research areas have been identified that need to be better accessed, especially while comparing the content and biological activities of different varieties of *Theobroma cacao*. The relevant “state of the art” also suggests that exploration of other potentially bioactive components in cocoa needs to be undertaken, while considering the complexity of reaction products occurring during the roasting phase of the post-harvest processing. In the same context, there is an increasing interest in two further involved compound classes (proteins and phenolics) from the chemical point of view, which is related directly or indirectly to their dual role as substrates for oxidative-monitored reactions, and integration of mass spectrometric methods may provide a valuable tool for their characterization. Finally, the utilization of partially processed cocoa beans (e.g., fermented, conciliatory thermal treatment) provides a large collection of bioactive potentials that could be included in the designing of functional foods, illuminating an alternative use of cocoa especially in the cocoa-producing countries to bolster the diets with corresponding positive impact on the health status of the local populations.

## Figures and Tables

**Table 1:** Dominant reported and postulated changes in the protein fractions during the seed maturation and post-harvest processing.

Determinants	Changes in Protein Fraction	Mechanisms Involved	Relevant Studies	Remarks
Genetic predisposition Location/climate soil/fertilization Stress conditions Maturation	Content and composition/Variation Post-transitional modification	Protein expression and accumulation/Phosphorylation/Glycosylation/Oxidation/Carbonylation	[28,29,32,43,44,62,63]	Reactive oxygen, carbonyl and nitrogen species that react with the proteins under stress conditions
Harvest/Storage, Pre-conditioning, Fermentation conditions (pH, temperature, method, and location/climate)	Degradation Post-transitional modification	Proteolytic processing/Phosphorylation/Glycosylation/Oxidation, Carbonylation, Deamidation, Decarboxylation Bound phenolics	[10,11,12,22,50,57]	Production of precursors for roasting—peptides/amino acids/reducing sugars/lipid degradation/Phenol modification
Drying, Roasting conditions (temperature, time, method etc.)	Thermal modification/Reactions with other constituents	Maillard reaction Volatile compounds/Aroma and flavor development/Browning/Covalent bound phenolics	[9,65,68,71,73,82,83]	600 flavor compounds [65,84] Melanoidin Fractions [86,87,88]
Further processing conditions/Alkalization	Interactions with phenolic compounds? [7,51]	Oxidation and polymerization of phenolic compounds/Reduced acidity/Improved sensory perception/quality	[65,71,85]	Browning/Melanoidin fractions [86,87,88]

**Table 2:** Selected examples for bioactivity potentials connected with cocoa protein fraction.

Protein-Related Fractions	Documented Bioactivity	Relevant Studies
Intact proteins, e.g., cysteine protease Albumin and glutelin fractions	Response of the action of the parasite *Moniliophthora perniciosa*, a fungus that causes witches’ broom	[56]
	Radical-scavenging capacity	[113,114]
Release of bioactive peptides during fermentation	Antioxidants—therapeutically interesting for the prevention of age-related diseases	[112]
	Antitumor activity in cell culture studies	[113]
	Anti-hypertensive activities	[115]
	Dipeptidyl peptidase IV inhibitory activity—anti-obesity and anti-hyperglycemic effects	[117]
Enzymatic and/or (chemical) decarboxylation of amino acids	Bioactive amines–contribution to antioxidant activity Depending on concentration—positive/adverse effects to human health	[118]
Thermal modification	Maillard reaction/Melanoidins—antioxidant, antimicrobial, anticancer, antihypertensive, cytotoxic, genotoxic, and detoxifying activities	[121,124]
	Interactions with phenolic compounds—antioxidant activity	[86,87,88]
Direct modification by phenolic compounds	Fermentation/Alkalization; retention of some of the polyphenol structure and activity and/or introduction of potentially new activities	[7,51,86,87,88,117]

## Supplementary Materials

References [126,127,128,129] are cited in the supplementary materials. The following are available online at https://www.mdpi.com/2072-6643/11/2/428/s1, Figure S1: Chronological order of the steps taken to produce the final model of the vicilin storage protein, Figure S2: Calculated and postulated phosphorylation sites (green) in the storage protein vicilin from *Theobroma cacao* (Entry: Q43358; 60.8 kDa/525 amino acids; reviewed version from the database UniProt-http://www.uniprot.org/; 28.01-2019). The data indicates that the reported modifications (orange) at positions 232 (Thr), 235 (Ser) and 240 (Ser), as reported in [50] are most probably occurring after the proteolytic processing of the 66-kDa precursor [9,22], Figure S3: Calculated and postulated glycosylation sites (O-GLcNAc; pink) in the storage protein vicilin from *Theobroma cacao* (Entry: Q43358; 60.8 kDa/525 amino acids; reviewed version from the database UniProt-http://www.uniprot.org/; 28.01-2019). The data indicates that the reported modifications at positions 193 (Thr), 235 (Ser), 338 (Thr) and 474 (Thr) as reported in [50] are most probably occurring after the proteolytic processing of the 66 kDa precursor [9,22], Table S1: Templates utilized for the homology model for vicilin from *Theobroma cacao*.
