# Supplementary material for: Cocoa Bean Proteins—Characterization, Changes and Modifications due to Ripening and Post-Harvest Processing
Source: Nutrients. 2019 Feb 19;11(2):428. doi: 10.3390/nu11020428 (PMC6413064; doi:10.3390/nu11020428)

Molecular modeling experiments

Molecular modeling experiments were performed using the following molecular modeling softwares: Molecular Operating Environment (MOE). Both MOE 2018.1 and Yasara 18.12.27 [126–128] were used for homology modeling of the vicilin storage globulin. The general methodology for the modeling of the vicilin storage protein is summarized in Figure S1. Details are given in the following studies [51,129]. The used templates for the homology model are given in Table S1. The amino acid sequence of the storage protein vicilin from *Theobroma cacao* (Entry: Q43358; 60.8 kDa/525 amino acids reviewed version from the database UniProt - http://www.uniprot.org/; 28.01-2019) was used for the modeling experiments and determination of the possible phosphorylation and glycosylation (O-GLcNAc) sites.

Table S1 Templates utilized for the homology model for vicilin from *Theobroma cacao*

Template	*Total score*	*BLAST E-value*	*Align score*	*Cover*	*ID*	*Resolution*	*Header*
1	380.14	5e-081	717.0	67%	5VF5-A	1.49 A	XRAY SM80.1 Vicilin [Solanum melongena] <NA> (370 residues with quality score 0.793), released 2017-12-06
2	352.24	3e-083	672.0	67%	5YJS-A	2.16 A	XRAY vicilin-like antimicrobial peptides 2-2 [Capsicum annuum] <NA> (370 residues with quality score 0.784), released 2018-10-10
3	346.19	1e-084	753.0	65%	5E1R-E	2.65 A	XRAY 7S vicilin [Carya illinoinensis] <B3STU4_CARIL(369-792)> (358 residues with quality score 0.712), released 2016-08-10
4	320.10	9e-108	604.0	68%	2EA7-C	1.80 A	XRAY 7S globulin-1 (Fragment) [Phaseolus angularis] <A4PI98_PHAAN(1-434)> (379 residues with quality score 0.778), released 2008-02-05
5	319.40	2e-076	612.0	69%	5CAD-A	1.49 A	XRAY SM80.1 Vicilin [Solanum melongena] <A0A158RFR1_SOLME(1-392)> (379 residues with quality score 0.759), released 2016-04-06

Figure S1 Chronological order of the steps taken to produce the final model of the vicilin storage protein.

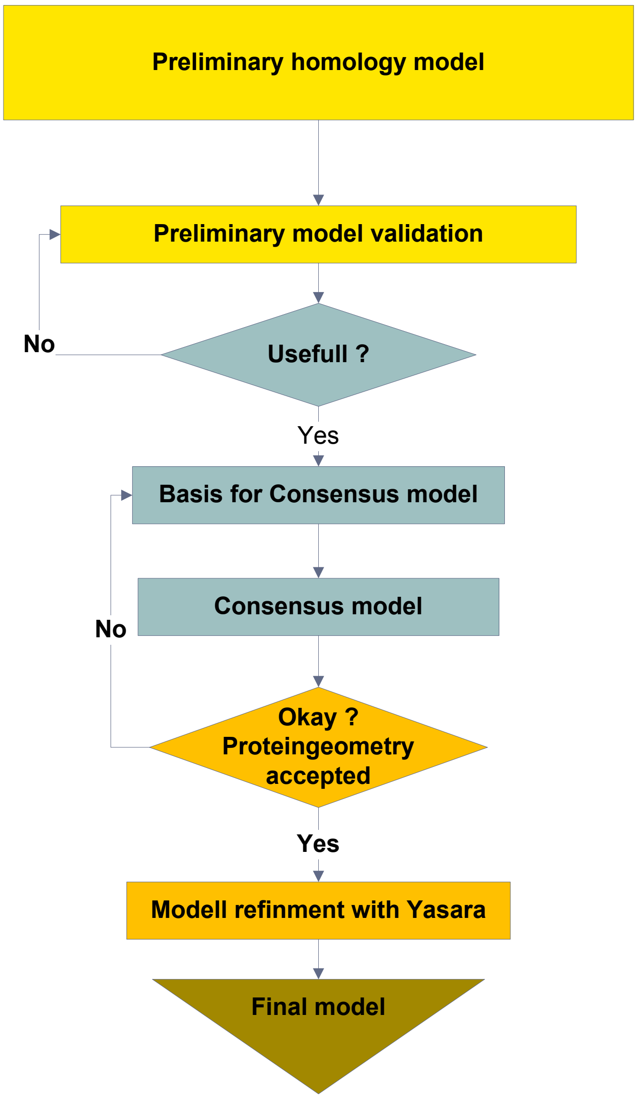

Figure S2: Calculated and postulated phosphorylation sites (green) in the storage protein vicilin from *Theobroma cacao* (Entry: Q43358; 60.8 kDa/525 amino acids; reviewed version from the database UniProt - http://www.uniprot.org/; 28.01-2019). The data indicates that the reported modifications (orange) at positions 232 (Thr), 235 (Ser) and 240 (Ser), as reported in [50] are most probably occurring after the proteolytic processing of the 66-kDa precursor [9,22].

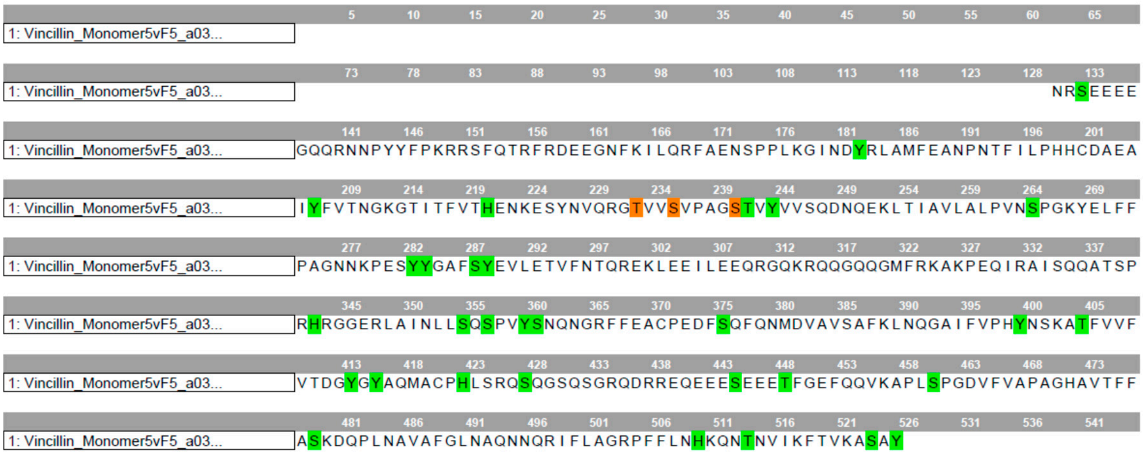

Figure S3: Calculated and postulated glycosylation sites (O-GLcNAc; pink) in the storage protein vicilin from *Theobroma cacao* (Entry: Q43358; 60.8 kDa/525 amino acids; reviewed version from the database UniProt - http://www.uniprot.org/; 28.01-2019). The data indicates that the reported modifications at positions 193 (Thr), 235 (Ser), 338 (Thr) and 474 (Thr) as reported in [50] are most probably occurring after the proteolytic processing of the 66-kDa precursor [9,22].

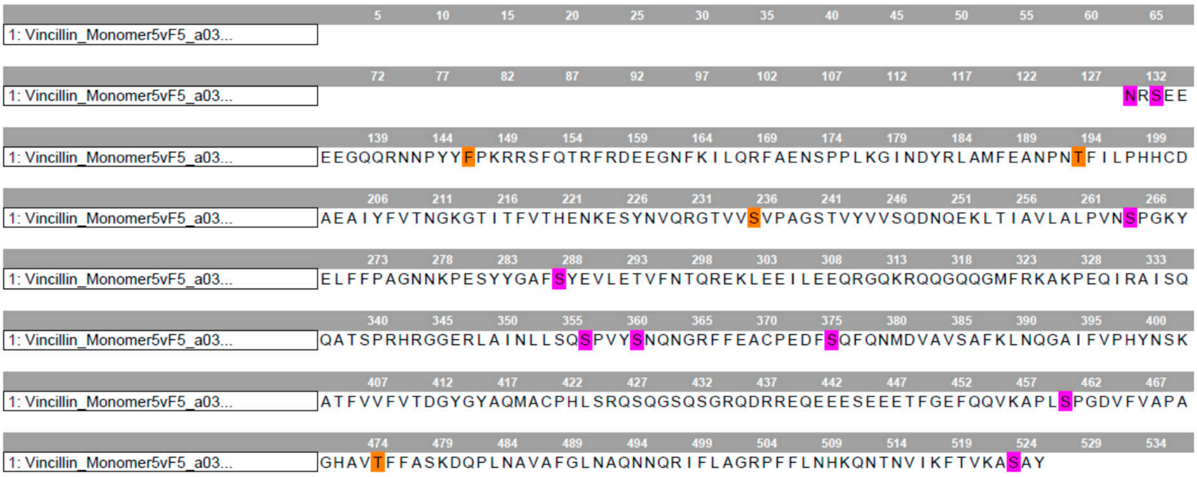

Supplement: Supplementary file 1 [file nutrients-11-00428-s001.pdf]
